# Correlation of CT-Derived Quantitative Image Features and Inflammatory Laboratory Markers with Length of Hospital Stay in Patients with Pyelonephritis

**DOI:** 10.3390/diagnostics16183044

**Published:** 2026-09-19

**Authors:** Markus Graf, Tristan Lemke, Alexander W. Marka, Nicolas Lenhart, Sebastian Ziegelmayer, Marcus R. Makowski, Stefan Reischl, Andreas Sauter, Keno K. Bressem, Lisa C. Adams, Thomas Huber

**Affiliations:** Department of Diagnostic and Interventional Radiology, Klinikum Rechts der Isar, School of Medicine and Health, Technical University of Munich, 81675 Munich, Germany; markus.m.graf@tum.de (M.G.); alexander.marka@tum.de (A.W.M.); nicolas.lenhart@tum.de (N.L.); s.ziegelmayer@tum.de (S.Z.); marcus.makowski@tum.de (M.R.M.); stefan.reischl@tum.de (S.R.); andreas.sauter@tum.de (A.S.); keno.bressem@tum.de (K.K.B.); lisa.adams@tum.de (L.C.A.); thomas-huber@tum.de (T.H.)

**Keywords:** acute pyelonephritis (APN), CT, quantitative imaging features, length of hospital stay (LOS)

## Abstract

**Background/Objectives:** To assess associations between quantitative computed tomography (CT) features, inflammatory markers, and length of hospital stay (LOS) in acute pyelonephritis (APN). **Methods:** This retrospective single-center study included 82 patients with CT-confirmed APN. Two radiologists quantified renal perfusion-deficit volume and percentage and perirenal fat-stranding (PFS) area and attenuation. Associations with C-reactive protein (CRP), white blood cell (WBC) count, procalcitonin, and LOS were assessed using Spearman correlation and regression analyses. Receiver operating characteristic (ROC) curve analysis evaluated LOS ≥ 10 days. **Results:** Perfusion-deficit volume and percentage correlated strongly with CRP (ρ = 0.763 and 0.714; both *p* < 0.001) and weakly with WBC count (ρ = 0.379 and 0.374; both *p* < 0.001). PFS area correlated moderately with procalcitonin (ρ = 0.482, *p* = 0.001; *n* = 42). Both perfusion-deficit measures correlated moderately with LOS (ρ = 0.538 and 0.536; both *p* < 0.001). In adjusted linear regression, CRP remained associated with LOS (Coefficient = 0.523 days per 10 mg/L; 95% CI, 0.345–0.692; *p* < 0.001), whereas perfusion-deficit percentage did not (*p* = 0.430). In adjusted logistic regression, CRP, age, and male sex were associated with LOS ≥ 10 days. The multivariable model yielded an AUC of 0.883 (95% CI, 0.81–0.95). Results were unchanged in a sensitivity analysis excluding the seven outpatients, and bootstrap internal validation yielded an optimism-corrected AUC of 0.86. **Conclusions:** Quantitative renal perfusion deficits were associated with inflammatory burden and LOS in univariable analyses. CRP showed the most consistent independent association, whereas the incremental value of CT metrics requires external validation.

## 1. Introduction

Acute Pyelonephritis (APN), a common infection of the renal pelvis and kidney, often occurs as a complication of a urinary tract infection (UTI) and can present significant diagnostic and management challenges in clinical practice [1]. Studies report a rising incidence of APN in hospitalized patients, and the clinical presentation varies from mild, uncomplicated cases to severe forms involving renal abscess or sepsis, making accurate diagnosis and grading crucial but challenging for effective treatment [2]. Unlike other common infections, the severity of APN is not always apparent from clinical examination alone [3], which necessitates reliable imaging and laboratory markers for comprehensive assessment [4,5].

Laboratory markers of inflammation, such as C-reactive protein (CRP), white blood cell (WBC) count, and procalcitonin, play an essential role in assessing APN [6,7]. These markers provide a biochemical snapshot of the systemic inflammatory response and can aid in prognosis and management decisions [1].

According to the American College of Radiology (ACR) Appropriateness Criteria, imaging studies, including computed tomography (CT), MRI, and ultrasound (US), are recommended primarily for high-risk patients with suspected APN to aid diagnosis and identify complications [8]. A systematic review of patients with APN found that while US is commonly used, it often reveals few abnormalities and infrequently leads to changes in clinical management, questioning the routine use of US in these cases [9]. Contrast-enhanced CT is the gold-standard imaging modality with a high accuracy in diagnosing APN [8,10], offering detailed visualization of renal anatomy and pathology. CT imaging can reveal key features such as renal enlargement, cortical loss, abscess formation, and areas of decreased perfusion [10,11,12,13]. Previous studies have highlighted the clinical potential of CT-derived quantitative image features to identify APN [14], which could be beneficial for guiding treatment decisions. Despite the recognized utility of CT and inflammatory markers, there is limited research on their combined use for predicting disease severity and outcomes, including the length of hospital stay (LOS). The aim of this study was to investigate the correlation between CT-derived quantitative image features, laboratory markers and LOS in patients with APN, to improve the understanding of APN and non-invasive assessment of its severity.

## 2. Methods

### 2.1. Patient Characteristics and Imaging Acquisition

The study was designed as a single-center retrospective study at our tertiary care institution. Patients with symptoms of APN who presented to our institution between December 2018 and April 2024 were assessed for study eligibility. This inclusion period was defined a priori, and the dataset was locked for statistical analysis after completion of the standardized dual-reader image analysis; patients presenting after April 2024 were therefore not part of the present analysis. Data collection, processing, and analysis were approved by the institutional review board (protocol number 180/17S, date of approval: 9 May 2017), and informed consent was waived. Inclusion criteria were as follows: (1) clinical symptoms of APN (including fever, flank pain, dysuria and/or pollakisuria), (2) infectious constellation in terms of elevated CRP and/or WBC obtained within the first 24 h after the CT examination, and (3) CT morphologic signs consistent with APN, such as hydronephrosis, perfusion deficit of the renal parenchyma, and/or perirenal fat stranding (PFS). In line with standard clinical practice for APN, hospitalization was primarily indicated by systemic symptoms such as fever and flank pain in combination with elevated inflammatory markers. Discharge decisions were made according to routine institutional clinical practice. Patients were considered eligible for discharge once they showed clinical improvement, absence of persistent fever, adequate pain control, and the ability to tolerate oral intake and/or oral antibiotic therapy. In addition, relevant complications such as abscess formation, obstruction, or need for intervention were considered before discharge. No cases with immunosuppression or ongoing antibiotic therapy prior to the diagnosis and CT examination were included. Exclusion criteria were (1) absence of an infectious constellation, (2) concomitant acute pathology (e.g., pancreatitis, cholecystitis or appendicitis), (3) absence of CT morphologic signs consistent with APN, and (4) insufficient image quality (e.g., motion artifacts). In total, 101 patients with clinical, biochemical and CT-morphological signs of APN met the initial screening criteria. Of these, 19 were excluded due to concomitant acute pathology (*n* = 14) and insufficient image quality (*n* = 5).

All patients underwent a standardized abdominal CT protocol. In this cohort, CT was indicated in cases of suspected complicated APN, severe or atypical clinical presentation, suspected urinary obstruction or abscess formation, or when exclusion of alternative acute abdominal pathology was required. Images were acquired in the venous contrast phase after intravenous application of iodinated contrast agent (Ultravist^®^-370, Bayer AG, Berlin, Germany, 80 mL, 3.5 mL/s) and bolus tracking with a region of interest (ROI) placed in the aorta. A soft tissue reconstruction kernel was used for multiplanar reconstruction.

### 2.2. Image Analysis

**Figure 1:**
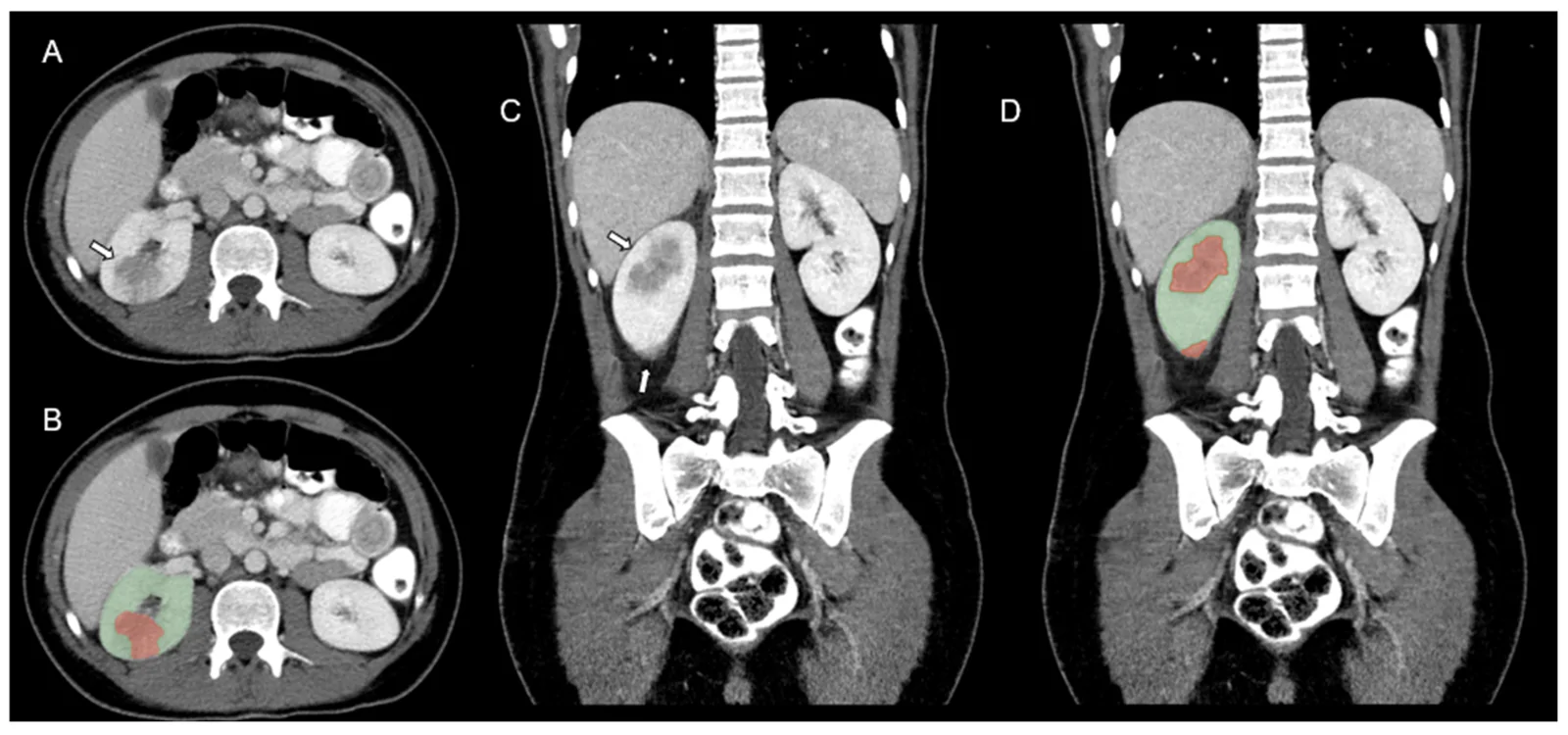
Contrast-enhanced computed tomography (CT) imaging identified multifocal perfusion deficits in the right kidney of a female patient, indicated by white arrows in the axial (**A**) and coronal (**C**) planes. Panels (**B**,**D**) show the corresponding segmentation overlays used to quantify the total volume of the affected kidney and the perfusion deficit, with the kidney segmentation shown in green and the perfusion deficit shown in red. The perfusion defect volume was 20.6 cm^3^, accounting for 12.3% of the total affected kidney volume of 168.0 cm^3^. The patient had a C-reactive protein (CRP) level of 202 mg/L and a white blood cell count (WBC) of 20.17 × 10^9^/L.

Two radiologists, each with over six years of experience, independently reviewed the CT images while blinded to the clinical and laboratory data. They measured the total volume of the affected kidney and renal perfusion deficits, as well as the maximum PFS area and average PFS density in the axial and coronal planes. For patients with bilateral APN (*n* = 7), the quantitative CT features were calculated by adding the measurements from both kidneys together. Analyses and segmentations were performed using a certified PACS workstation (IDS7 21.2; Sectra, Linköping, Sweden). Renal perfusion deficits were manually segmented on contrast-enhanced venous phase CT images by delineating visually hypoenhancing parenchymal areas consistent with APN. Total kidney volume was obtained by manual segmentation of the affected renal parenchyma, and the perfusion deficit percentage was calculated as the ratio of perfusion deficit volume to total kidney volume. PFS area was measured manually at the level of maximum perirenal inflammatory change, while average PFS density was assessed by measuring the density of the entire PFS area. Additionally, the average density of intra-abdominal fat was measured by placing a 10 mm region of interest on the contralateral side of Gerota’s fascia. Lymphadenopathy was defined as lymph nodes with a short-axis diameter > 10 mm on CT. Figure 1 illustrates an example perfusion deficit segmentation.

For all continuous CT-derived quantitative features, including perfusion-deficit volume, perfusion-deficit percentage, PFS area, and PFS density, the mean of the two readers’ measurements was used for statistical analysis.

### 2.3. Laboratory Markers

Laboratory values were obtained from the hospital’s laboratory information system on the day of the CT scan. The following laboratory markers were recorded for analysis: creatinine, CRP, WBC count, and procalcitonin. Procalcitonin values were available for 42 patients (51.2%) on the day of the CT scan. Throughout the manuscript, CRP values are reported in mg/L, WBC counts in ×10^9^/L, and procalcitonin values in ng/mL.

### 2.4. Study Outcomes

The primary outcome was the association between CT-derived quantitative imaging features (perfusion-deficit volume, perfusion-deficit percentage, PFS area, and PFS density) and inflammatory laboratory markers (CRP, WBC count, and procalcitonin). Secondary outcomes were (1) the association of imaging features, laboratory markers, and demographic parameters with LOS as a continuous variable and (2) their ability to discriminate prolonged hospitalization, defined as LOS ≥ 10 days (median split), including the discriminative performance of a multivariable model combining imaging, laboratory, and demographic parameters.

### 2.5. Statistical Analysis

Statistical analyses were performed using Python (version 3.8), including SciPy (version 1.10.1), statsmodels (version 0.14.0) and scikit-learn (version 1.2.2). Intraclass correlation coefficients (ICCs) were calculated for each imaging feature to assess inter-reader agreement. As excellent agreement was observed for all measurements (ICC: PFS area > 0.89, PFS density > 0.87 and perfusion deficit volume > 0.92), the mean values of the two readers were used. The distributions of continuous variables were assessed using the Shapiro–Wilk test. As most variables, including CRP, WBC count, procalcitonin, LOS, and the CT-derived quantitative features, deviated significantly from a normal distribution (*p* < 0.05), non-parametric tests were applied and descriptive statistics are reported as median [interquartile range, IQR]. Spearman’s rank correlation was used to evaluate the association between continuous imaging features (perfusion deficit volume and percentage, and PFS area and density) and continuous laboratory values (CRP, WBC and procalcitonin). Mann–Whitney U tests were used to compare laboratory values between groups defined by categorical imaging findings. Finally, univariate linear regression models were used to quantify the ability of CT features to predict CRP, WBC, procalcitonin and LOS. Additionally, multivariable linear regression was performed to evaluate independent predictors of LOS as a continuous outcome. For binary outcome analyses (LOS ≥ 10 days, median split), we fitted univariate and multivariable logistic regression models. Age and sex were included as covariates in the multivariable models to account for potential confounding factors. Variance inflation factors (VIFs) were calculated to assess potential multicollinearity between predictors, with values greater than 5 indicating problematic collinearity. As a sensitivity analysis, all LOS-related regression models were repeated after excluding the seven patients managed on an outpatient basis (LOS = 0 days). Receiver operating characteristic (ROC) curve analyses were conducted to evaluate the ability of significant predictors and the multivariable logistic regression model to discriminate. The internal validity of the multivariable logistic model was assessed by bootstrap-based internal validation (1000 resamples): the model was refitted in each bootstrap sample, the optimism was estimated as the mean difference between the bootstrap and original-sample AUC, and an optimism-corrected AUC was derived by subtracting this estimate from the apparent AUC; as a supplementary check, stratified five-fold cross-validation with 200 repetitions was performed, with the AUC averaged across all held-out folds. The area under the curve (AUC) and optimal cut-offs based on the Youden index were reported. The strength of correlation coefficients was interpreted according to the following criteria: very weak (0.00–0.19), weak (0.20–0.39), moderate (0.40–0.59), strong (0.60–0.79), and very strong (0.80–1.00) [15]. A *p*-value of less than 0.05 was considered statistically significant.

## 3. Results

### 3.1. Patient Characteristics

After exclusion criteria, the final study cohort included a total of 82 patients with a median age of 61.7 years [IQR, 48.9–76.4; range, 18–91].

The gender distribution was 64.6% male and 35.4% female. Inflammatory markers were elevated, with a median CRP level of 153 mg/L [IQR, 74–256] and a median WBC count of 12.7 × 10^9^/L [IQR, 10.1–17.3]. Procalcitonin, available for 42 patients, had a median level of 3.3 ng/mL [IQR, 0.4–17.4]. Of 82 patients, 75 (91.5%) were hospitalized and 7 (8.5%) managed on an outpatient basis (LOS = 0). The median LOS was 10 days [IQR, 6–18.8; range, 0–31]. Complications on CT included renal abscesses in 12.2% and intrarenal gas in 2.4%, while 67.1% showed locoregional lymphadenopathy. The baseline imaging features included a median PFS area of 902 mm^2^ [IQR, 531–1380] and a median perfusion-deficit volume of 20.3 cm^3^ [IQR, 7.1–43.8]. Detailed patient characteristics and imaging metrics are shown in Table 1.

### 3.2. Biochemical Correlation with CT-Derived Quantitative Image Features

#### 3.2.1. CRP

Spearman correlation analysis revealed a strong positive, significant correlation (ρ = 0.76, *p* < 0.001) between CRP levels and the total volume of renal perfusion deficit. Another strong, positive, significant correlation (ρ = 0.71, *p* < 0.001) was observed between CRP levels and perfusion deficit as a percentage of total kidney parenchyma. In contrast, the analysis found a very weak positive correlation (ρ = 0.12, *p* = 0.29) between CRP levels and the average area PFS.

**Figure 2:**
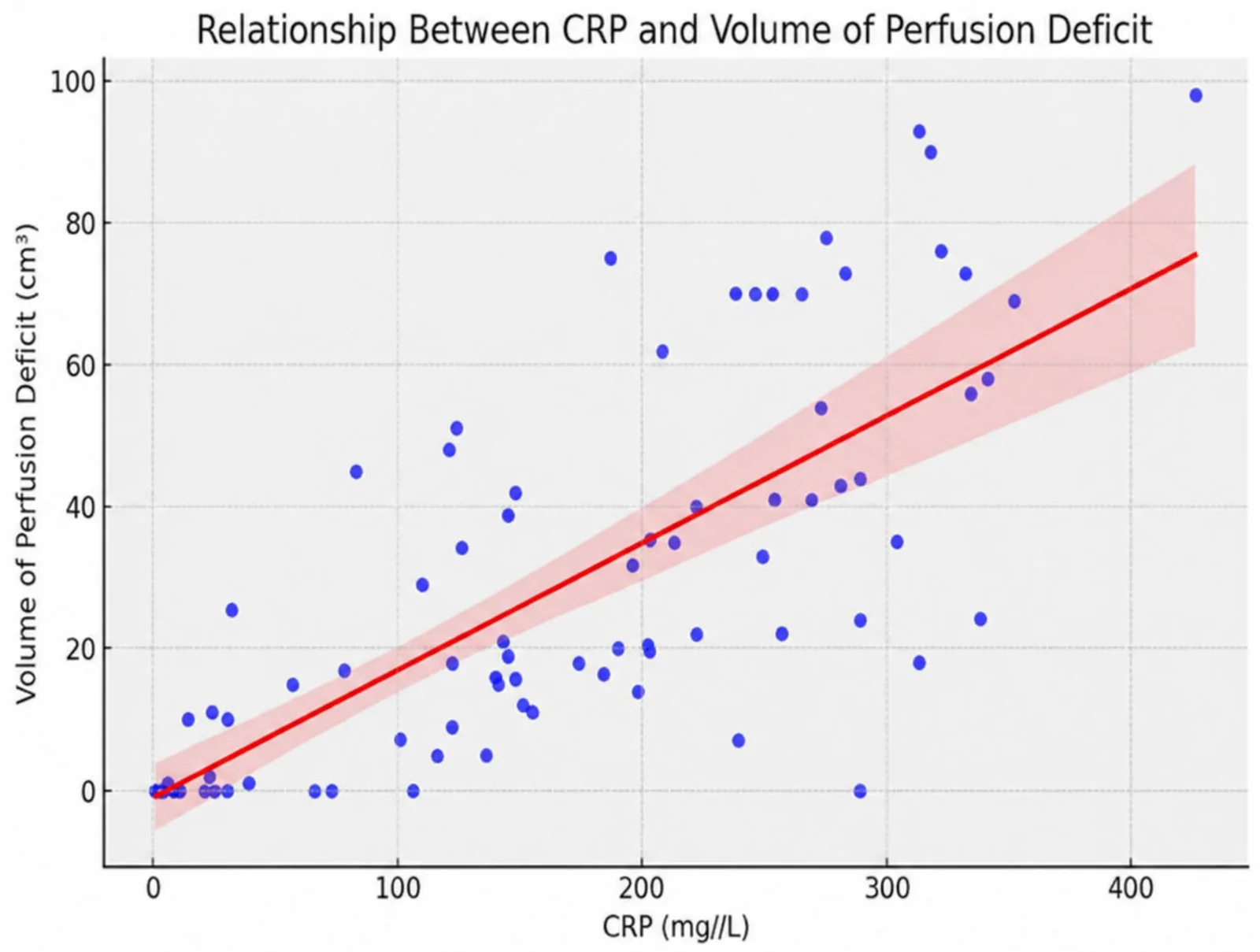
This scatterplot illustrates the relationship between the volume of renal perfusion deficit [cm^3^] and CRP levels [mg/L]. The red line represents the linear regression fit; the shaded area indicates the 95% confidence interval.

In univariable linear regression, perfusion-deficit volume and percentage were significant predictors of CRP (R^2^ = 0.545 and R^2^ = 0.435, respectively; both *p* < 0.001), corresponding to an increase in CRP of approximately 3.0 mg/L per cm^3^ of perfusion-deficit volume and 6.3 mg/L per percentage point of perfusion deficit (Table 2). Figure 2 illustrates the significant positive correlation between CRP levels and the volume of renal perfusion deficit, demonstrating the linear relationship described above.

There were no significant differences in CRP levels between patients with and without abscess formation (*p* = 0.989) or renal gas (*p* = 0.833). However, patients with locoregional lymphadenopathy had significantly higher CRP levels than those without (*p* = 0.0017).

#### 3.2.2. WBC

The WBC count showed weak positive correlations with both perfusion deficit volume (ρ = 0.379, *p* < 0.001) and perfusion deficit percentage (ρ = 0.374, *p* < 0.001). A weaker, yet still significant, correlation was observed between WBC and PFS area (ρ = 0.222, *p* = 0.046). In contrast, PFS density showed no meaningful correlation (ρ = −0.027, *p* = 0.812). Consistent with the CRP results, there was no significant difference in WBC counts based on the presence of an abscess (*p* = 0.590) or renal gas (*p* = 0.714). However, WBC counts were higher in patients with locoregional lymphadenopathy (*p* = 0.041). In univariable linear regression, perfusion-deficit volume and percentage were significant predictors of the WBC count, whereas PFS area and density had minimal explanatory power (Table 2).

#### 3.2.3. Procalcitonin

Procalcitonin data were only available for 42 patients. No significant correlations were found between procalcitonin levels and most computed tomography (CT) features. However, perfusion deficit volume (ρ = 0.201, *p* = 0.208) and perfusion deficit percentage (ρ = 0.232, *p* = 0.144) exhibited weak, non-significant positive correlations with procalcitonin. PFS density showed no correlation (ρ = −0.059, *p* = 0.713). However, procalcitonin exhibited a moderate positive correlation with PFS area (ρ = 0.482). Nevertheless, this was based on a limited sample, so its significance (*p* = 0.0014) should be interpreted with caution. There was also no significant difference in procalcitonin levels between patients with and without abscess (*p* = 0.954), gas (*p* = 0.927) or lymphadenopathy (*p* = 0.137). Likewise, linear regression did not yield any significant predictive relationship between procalcitonin and the imaging features. Given that procalcitonin was available in only 42 of 82 patients, these analyses were likely underpowered, and the absence of significant correlations should be interpreted with caution. The complete univariable linear regression results for the associations of CT-derived features with CRP, WBC count, and procalcitonin are summarized in Table 2.

### 3.3. Correlation of CT-Derived Features, Inflammatory Laboratory and LOS

A moderate positive correlation was observed between LOS and perfusion deficit volume (ρ = 0.538, *p* < 0.001), as well as between LOS and perfusion deficit percentage (ρ = 0.536, *p* < 0.001). Therefore, patients with larger perfusion deficits tended to have longer hospital stays. In contrast, PFS area (ρ = 0.114, *p* = 0.311) and PFS density (ρ = 0.128, *p* = 0.256) showed no significant correlation with LOS. In linear regression models, perfusion deficit volume and percentage were significant predictors of LOS, explaining approximately 24% (R^2^ = 0.242) and 23% (R^2^ = 0.230) of the variance in LOS, respectively (*p* < 0.001 for both). PFS features had negligible predictive value (R^2^ < 0.02, *p* > 0.25). Among the laboratory markers, CRP showed a strong positive correlation with LOS (ρ = 0.649, *p* < 0.001) and was a significant univariable predictor of LOS (R^2^ = 0.416, *p* < 0.001). WBC count (ρ = 0.231, *p* = 0.037) and procalcitonin (ρ = 0.446, *p* = 0.003; *n* = 42) correlated with LOS, but showed only non-significant trends in univariable linear regression. Age and sex were not significantly associated with LOS in univariable analyses. The complete univariable regression results are summarized in Table 3.

In a multivariable regression model including perfusion deficit percentage and CRP, the overall explained variance was R^2^ = 0.410. CRP remained significantly associated with LOS (Coefficient = 0.480 days per 10 mg/L; *p* < 0.001), whereas perfusion deficit percentage was not significant (Coefficient = 0.060 days per 1 percentage-point increase; *p* = 0.499). After additional adjustment for age and sex, the explained variance increased to R^2^ = 0.501. In this fully adjusted model, CRP remained a strong independent predictor of LOS (Coefficient = 0.523 days per 10 mg/L; *p* < 0.001), while age (Coefficient = 0.102; *p* = 0.013) and male sex (Coefficient = +3.38; *p* = 0.034) were also independently associated with longer LOS. Perfusion deficit percentage did not reach statistical significance in the adjusted model (Coefficient = 0.066 days per 1 percentage-point increase; *p* = 0.430). Variance inflation factors (VIFs) were calculated for all predictors. VIF values were 1.74 for perfusion deficit percentage, 1.78 for CRP, 1.07 for age, and 1.06 for sex, indicating no problematic multicollinearity. The univariable and multivariable linear regression results for LOS are summarized in Table 3.

Predictors of prolonged hospitalization were evaluated with a univariate logistic regression analyses using LOS ≥ 10 days (median split) as the binary outcome. Among CT-derived features, a higher perfusion deficit percentage was significantly associated with a prolonged LOS (OR = 1.11 per 1 percentage-point increase, *p* < 0.001), as was a higher perfusion deficit volume (OR = 1.03, *p* = 0.002). However, PFS area (OR = 1.00, *p* = 0.348) and PFS density (OR = 1.00, *p* = 0.985) were not related to LOS of at least 10 days. Regarding inflammatory laboratory markers, C-reactive protein (CRP) was significantly associated with prolonged LOS (OR = 1.11, *p* < 0.001), whereas white blood cell (WBC) count (OR = 1.05, *p* = 0.125) and procalcitonin (OR = 1.01, *p* = 0.354) were not significant predictors. Among demographic parameters, age was significantly associated with a longer LOS (OR = 1.03, *p* = 0.011), while male sex showed a nonsignificant trend toward higher odds of a prolonged LOS (OR = 2.30, *p* = 0.082).

To investigate independent predictors of prolonged hospitalization, a multivariable logistic regression analysis was performed using LOS ≥ 10 days as the outcome. In a model including perfusion deficit percentage and CRP, only CRP remained significantly associated with prolonged LOS (OR = 1.07 per 10 mg/L increase, *p* = 0.031), while perfusion deficit percentage did not reach statistical significance (OR = 1.06 per 1 percentage-point increase, *p* = 0.079). After additional adjustment for age and sex, CRP (OR = 1.11 per 10 mg/L increase, *p* = 0.010), age (OR = 1.06 per 1-year increase, *p* = 0.002) and male sex (OR = 4.19, *p* = 0.044) were independently associated with prolonged LOS, whereas perfusion deficit percentage showed only a borderline association (OR = 1.09 per 1 percentage-point increase, *p* = 0.059). Variance inflation factors confirmed the absence of problematic multicollinearity (CRP 1.78, perfusion deficit % 1.74, age 1.07, sex 1.06). The univariable and multivariable logistic regression results for LOS ≥ 10 days are summarized in Table 4.

To evaluate the ability of clinical and imaging predictors to discriminate prolonged hospitalization, receiver operating characteristic (ROC) curve analyses were performed using a binary outcome of length of stay (LOS) of at least 10 days.

Univariate models revealed that CRP (AUC = 0.78 (95% CI, 0.67–0.88), optimal cut-off 0.38, sensitivity 0.93, and specificity 0.54) and perfusion deficit percentage (AUC = 0.77 (95% CI, 0.66–0.87), optimal cut-off 0.52, sensitivity 0.65, and specificity 0.79) were good discriminators. In contrast, age demonstrated only moderate discriminatory capacity (AUC = 0.67 (95% CI, 0.56–0.79), optimal cutoff 0.60, sensitivity 0.52, and specificity 0.79), and male sex yielded weak discrimination (AUC = 0.59 (95% CI, 0.49–0.69), optimal cutoff 0.66, sensitivity 0.44, and specificity 0.74).

**Figure 3:**
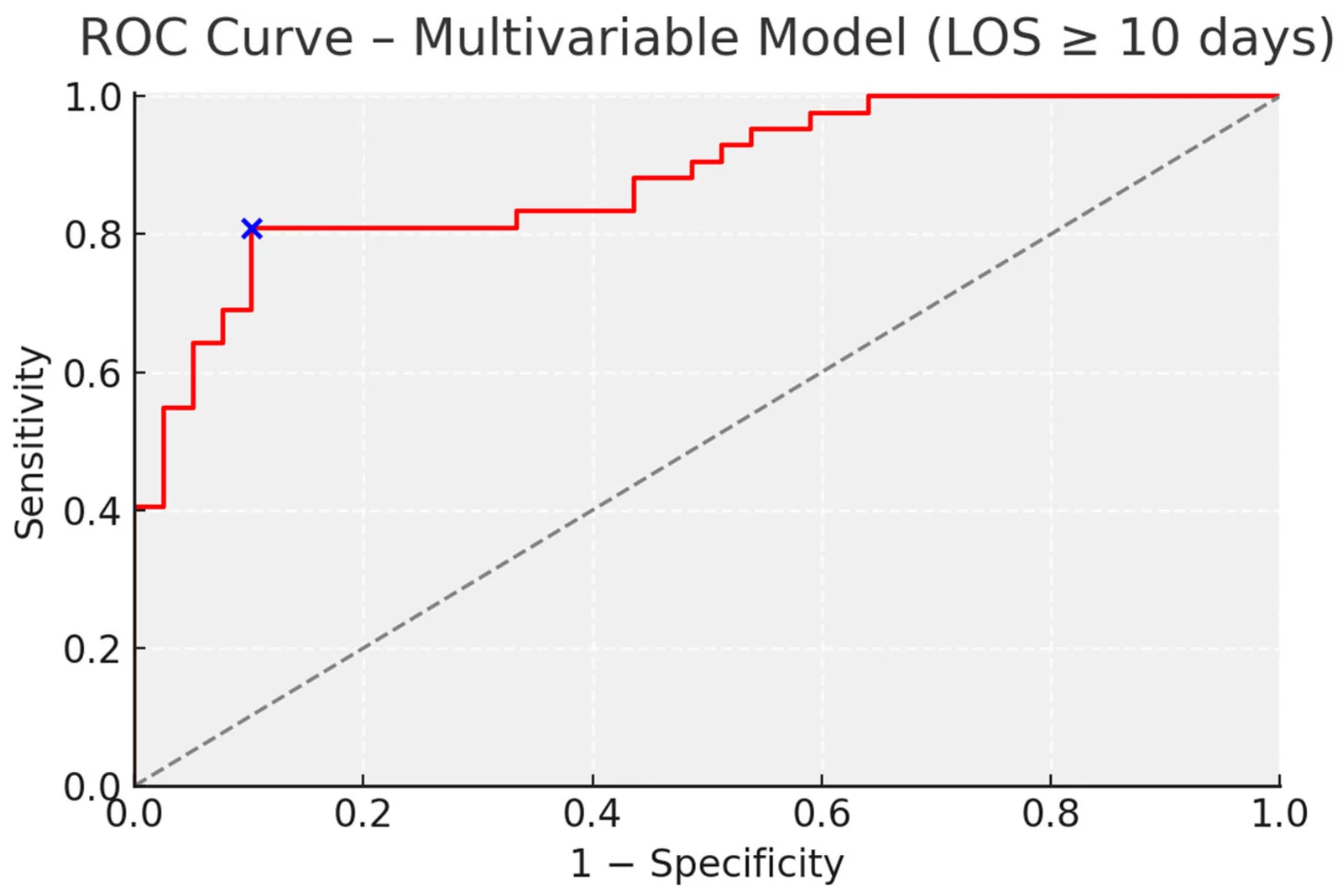
Receiver operating characteristic (ROC) curve of the multivariable logistic regression model (including CRP, perfusion deficit percentage, age, and sex) for prediction of prolonged hospitalization (LOS ≥ 10 days). The model achieved an AUC of 0.883 with an optimal cut-off of 0.62, resulting in a sensitivity of 0.81 and a specificity of 0.90. The dashed line indicates chance level; the blue marker denotes the optimal cut-off according to the Youden index.

The multivariable model including CRP, perfusion deficit percentage, age, and sex showed good apparent discrimination, with an AUC of 0.883 (95% CI, 0.81–0.95). The optimal cut-off (0.62) yielded a sensitivity of 0.81 and a specificity of 0.90. In the bootstrap-based internal validation, the estimated optimism was small (0.019), yielding an optimism-corrected AUC of 0.86; repeated five-fold cross-validation gave a comparable estimate (mean AUC = 0.86), indicating only modest overfitting. The ROC curve of the multivariable logistic regression model is shown in Figure 3.

### 3.4. Sensitivity Analysis Excluding Outpatients

In the sensitivity analysis excluding the seven patients managed on an outpatient basis (LOS = 0 days; *n* = 75), the pattern of results was unchanged. CRP remained independently associated with LOS in the fully adjusted linear model (coefficient = 0.47 days per 10 mg/L; *p* < 0.001), whereas perfusion-deficit percentage did not reach significance (*p* = 0.54). In the corresponding multivariable logistic regression, CRP (OR = 1.10 per 10 mg/L; *p* = 0.023), age (OR = 1.06 per 1-year increase; *p* = 0.003), and male sex (OR = 4.49; *p* = 0.039) remained independently associated with LOS ≥ 10 days, whereas perfusion-deficit percentage did not (OR = 1.08 per 1 percentage-point increase; *p* = 0.066); the model achieved an AUC of 0.86 (95% CI, 0.77–0.93).

## 4. Discussion

Our study highlights that CT imaging—beyond its diagnostic role in APN—can also help assess disease severity. We found that larger renal perfusion deficits on CT scans were strongly associated with higher levels of inflammatory markers (especially CRP) and longer LOS. This suggests that, alongside traditional laboratory tests, CT provides valuable prognostic information in APN.

Renal perfusion deficits in the course of APN can be a result of edema, vascular compression, microthrombi formation and subsequent obstructions in small renal vessels, or an effect of inflammatory mediators and cytokines [10,16,17]. CRP is an acute-phase protein that typically increases in response to inflammatory and infectious processes such as APN, just like WBC counts. The association between elevated CRP levels and compromised organ perfusion has been reported for different organs and pathologies. For instance, it was shown that higher CRP levels are associated with the extent of stress-induced myocardial ischemia [18], lung perfusion deficits in acute pulmonary infections [19] or necrotic areas in acute pancreatitis, which result from compromised organ perfusion [20]. This aligns with our findings, where both the volume and percentage of renal perfusion deficits show a positive correlation with elevated CRP levels, highlighting the diagnostic significance of these CT-derived parameters. The importance of CRP and WBC counts and their correlation with CT findings are consistent with the multimodal diagnostic approach emphasized in recent guidelines, which recommend a combination of clinical, laboratory, and imaging evaluations for the diagnosis of APN [8].

Fat stranding is a common sign in CT imaging of acute abdominal infections. Previous studies on different intraabdominal pathologies describe the presence of fat stranding being associated with the severity of inflammation in colitis [21], appendicitis [22] and pancreatitis [23]. However, our study showed less significant associations of CRP and WBC count with PFS. This aligns with findings from Fukami et al., showing that PFS is not a reliable diagnostic marker for APN as it shows no significant correlation with the inflammatory markers [24]. Quantifying PFS on CT scans may be technically challenging and can be affected by factors such as post-infectious changes or minimal stranding. This may explain the lack of correlation observed in our study.

Notably, we observed no meaningful relationship between procalcitonin levels and CT features of APN in our cohort. However, only 42 patients had procalcitonin measurements, which may have limited our ability to detect significant correlations. Given the reduced sample size and the correspondingly limited statistical power, the absence of significant correlations must be interpreted with caution and should not be regarded as evidence against an association between procalcitonin and imaging severity. Moreover, procalcitonin is itself a non-specific inflammatory marker whose routine measurement is associated with additional costs, and its incremental value over CRP in APN remains to be established. This conclusion appears to diverge from current studies discussing procalcitonin as a potential marker of infection severity [25,26,27]. Further investigations with larger, more comprehensive datasets are necessary to clarify these findings.

The observed correlation between CT-based features and LOS in APN has important clinical implications. Hospital LOS is a critical metric for patient care planning and resource allocation [28]. Therefore, having imaging markers that predict LOS can directly support clinical decision making. Identifying on CT that a patient has an extensive renal perfusion deficit—which our data associate with higher CRP level and longer LOS—could alert clinicians to a potentially serious course requiring intensive therapy or closer monitoring. However, these results should be interpreted with caution. Statistically, the correlation (ρ ≈ 0.54) indicates a moderate relationship, explaining only about a quarter of the variance in LOS. This underlines the multifactorial nature of LOS [28]. Although LOS is clinically relevant and may reflect disease severity, it is not determined by disease burden alone. LOS can also be influenced by non-clinical factors, including institutional discharge practices, physician decision-making, availability of outpatient care, social circumstances, and logistical factors. In addition, healthcare-system characteristics such as reimbursement schemes, bed availability, and local discharge culture differ substantially between institutions and countries and are well-established determinants of LOS [28,29]. Accordingly, the associations reported here partly reflect institution-specific practice patterns, and absolute LOS values and model coefficients should not be transferred directly to other healthcare settings. As no prospectively standardized discharge criteria were enforced in this retrospective cohort, LOS should be interpreted as a pragmatic clinical outcome rather than a direct or exclusive measure of APN severity. Notably, both perfusion deficit and CRP correlated with LOS in univariate analyses. However, our multivariable linear regression identified CRP as the most consistent independent predictor, whereas perfusion deficit lost statistical significance when adjusted for age and sex. This suggests that CRP, a routinely available laboratory marker, may capture much of the prognostic information reflected by CT perfusion deficits. Nevertheless, the consistent univariable associations suggest that quantitative CT metrics should be regarded as complementary to, rather than competing with, laboratory markers. In the population studied here, CT is typically performed as part of the initial diagnostic work-up of suspected complicated APN [8]; quantifying perfusion deficits therefore provides additional severity information without additional radiation exposure or examination costs. In contrast to CRP, which reflects the magnitude of the systemic inflammatory response in a non-specific manner [17], perfusion-deficit measurements localize and quantify the extent of renal parenchymal involvement, can reveal complications requiring intervention, and may be informative in situations in which CRP is difficult to interpret, for example, very early in the disease course before CRP has peaked. Based on the present data, however, quantitative CT metrics should currently be considered descriptive severity correlates and hypothesis-generating rather than independent predictors that outperform routinely available laboratory markers. ROC curve analysis showed that CRP and perfusion deficit percentage, when considered individually, provided good discriminatory ability for prolonged LOS (AUC ≈ 0.77–0.78). The multivariable model including CRP, perfusion deficit percentage, age, and sex showed good apparent discrimination in the present cohort, with an AUC of 0.883. These findings support the potential value of combining imaging and clinical parameters for risk stratification, while the incremental contribution of CT-derived parameters beyond routinely available clinical and laboratory variables requires further validation.

As our sample size was limited, particularly for procalcitonin, the ROC-curve-derived cut-offs and predictive estimates should be interpreted cautiously and warrant validation in larger, independent cohorts. In particular, the predictive models were developed and evaluated in the same small single-center cohort without external validation. Although bootstrap-based internal validation indicated only modest optimism (optimism-corrected AUC = 0.86), in accordance with established recommendations for prediction-model studies the reported estimates may still overestimate the discriminative performance achievable in independent populations and should be regarded as exploratory rather than as validated clinical decision thresholds [30]. Because we required CT-confirmed features consistent with APN, our study likely over-represents patients with more severe or complicated cases. In standard clinical practice, uncomplicated APN is often diagnosed clinically and treated without cross-sectional imaging, whereas CT is more commonly performed in patients with suspected complicated disease. Therefore, the indication for CT itself may represent a confounding factor, as patients undergoing CT may already have a higher pre-test probability of complicated disease, stronger inflammatory response, and longer hospitalization. This introduces a potential selection bias and limits the generalizability of our findings to milder cases that did not undergo CT imaging. Our cohort therefore represents the more severe end of the APN spectrum, and our findings cannot be extrapolated to uncomplicated APN, which is frequently diagnosed clinically and successfully managed without cross-sectional imaging; any potential clinical application of the proposed quantitative CT metrics would consequently be restricted to the subgroup of patients in whom CT is clinically indicated. Furthermore, comorbidities, concomitant infections during hospitalization, and microbiological data, including the causative organisms and the distinction between monomicrobial and polymicrobial infections, were not systematically recorded in this imaging-focused dataset; as comorbidity burden is a known determinant of prognosis and LOS, residual confounding cannot be excluded. Finally, in keeping with the a priori defined study period, patients treated after April 2024 were not included; this consecutive, more recent patient group represents a natural independent cohort for the future validation of our findings. Future prospective studies with larger, multicentre cohorts should therefore validate and refine these associations between imaging and outcomes, ideally incorporating multivariate analyses to control for confounders. Despite these limitations, our findings open up promising avenues for advanced AI-driven image analysis in APN. Radiomics and deep learning approaches could use the identified CT features (such as perfusion deficit volume) to build predictive models for individual LOS.

In conclusion, our findings support the complementary use of CT imaging and laboratory markers such as CRP for the severity assessment of patients with suspected complicated APN. While CRP was the strongest independent predictor of LOS, quantitative renal perfusion-deficit metrics provided consistent univariable severity information at no additional examination cost. Their incremental clinical value beyond routinely available laboratory markers, as well as the reported prediction estimates, require confirmation in larger, ideally multicenter cohorts with external validation.

## Figures and Tables

**Table 1:** Patient characteristics.

Characteristics	Value ^1^
Clinical characteristics	
Patients, *n*	82
Age, years	61.7 [48.9–76.4]
Sex (Male/Female)	53 (64.6%)/29 (35.4%)
Hospitalized patients	75 (91.5%)
Length of hospital stay, days	10 [6–18.8]
Laboratory findings	
C-reactive protein, mg/L	153.0 [74.3–256.3]
White blood cell count, 10^9^/L	12.7 [10.1–17.3]
Procalcitonin ^2^, ng/mL	3.25 [0.43–17.4]
Creatinine, µmol/L	88.4 [61.9–114.9]
CT findings	
Abscess formation	10 (12.2%)
Renal gas inclusion	2 (2.4%)
Locoregional lymphadenopathy	55 (67.1%)
Perirenal fat-stranding area, mm^2^	902 [531–1380]
Perirenal fat-stranding density, HU	−32.5 [−44.5 to −13.5]
Renal perfusion-deficit volume, cm^3^	20.3 [7.1–43.8]
Total affected kidney volume, cm^3^	196.0 [154.5–253.2]
Perfusion deficit, % of total kidney parenchyma	12.0 [4.3–21.0]

^1^ Data are presented as median [interquartile range] or *n* (%). ^2^ Procalcitonin was available in 42 patients. CT, computed tomography; HU, Hounsfield units.

**Table 2:** Combined linear regression analyses of inflammatory laboratory markers and computed tomography-derived features.

A. C-reactive protein (CRP)			
CT Feature	R^2^ (*p*)	Coefficient	Intercept
Area of PFS	0.011 (0.348)	0.015	152.06
Density of PFS	0.000 (0.957)	0.035	166.90
Volume of Perfusion Deficit	0.545 (<0.001)	3.040	78.86
Perfusion Deficit as a Percentage of Total Kidney Parenchyma	0.435 (<0.001)	6.271	80.98
B. White blood cell (WBC) count			
CT Feature	R^2^ (*p*)	Coefficient	Intercept
Area of PFS	0.028 (0.134)	0.0016	12.3522
Density of PFS	0.001 (0.822)	−0.0058	11.4519
Volume of Perfusion Deficit	0.144 (0.001)	0.1597	10.4162
Perfusion Deficit as a Percentage of Total Kidney Parenchyma	0.190 (<0.001)	0.1254	10.5107
C. Procalcitonin			
CT Feature	R^2^ (*p*)	Coefficient	Intercept
Area of PFS	0.020 (0.377)	0.0145	13.3858
Density of PFS	0.001 (0.819)	0.1087	53.2837
Volume of Perfusion Deficit	0.043 (0.191)	−0.5812	51.7867
Perfusion Deficit as a Percentage of Total Kidney Parenchyma	0.039 (0.206)	0.4604	43.8904

CRP, C-reactive protein; CT, computed tomography; PFS, perirenal fat stranding; WBC, white blood cell. CRP is expressed in mg/L; regression coefficients and intercepts of the CRP models are given in mg/L.

**Table 3:** Combined univariable and multivariable linear regression analyses for length of hospital stay (LOS).

A. Univariable linear regression analysis			
Feature	R^2^ (*p*)	Coefficient	Intercept
Area of PFS	0.008 (0.422)	0.0010	11.1968
Density of PFS	0.016 (0.256)	0.0593	13.9912
Volume of Perfusion Deficit	0.242 (<0.001)	0.1653	7.5055
Perfusion Deficit as a Percentage of Total Kidney Parenchyma	0.230 (<0.001)	0.3736	7.1956
CRP	0.416 (<0.001)	0.5256	3.4348
WBC	0.042 (0.064)	0.2399	8.8101
Procalcitonin	0.081 (0.069)	0.0316	14.5194
Age	0.018 (0.229)	0.0646	8.1829
Sex (male vs. female)	0.025 (0.160)	2.934	11.170
B. Multivariable linear regression analysis			
Feature	Coefficient	Intercept	*p*-Value
Intercept	n/a	−4.7788	0.101
CRP	0.523	n/a	<0.001
Perfusion Deficit as a Percentage of Total Kidney Parenchyma	0.066	n/a	0.430
Age	0.102	n/a	0.013
Male sex	3.380	n/a	0.034

Model R^2^ = 0.501. CRP, C-reactive protein; LOS, length of hospital stay; PFS, perirenal fat stranding; WBC, white blood cell; n/a, not applicable. Regression coefficients for CRP are expressed per 10 mg/L (equivalent to 1 mg/dL). Regression coefficients for perfusion deficit percentage are expressed per 1 percentage-point increase.

**Table 4:** Combined univariable and multivariable logistic regression analyses of predictors of LOS ≥ 10 days.

A. Univariable logistic regression analysis		
Feature	OR (95% CI)	*p*-Value
Area of PFS	1.00 (0.9997–1.0008)	0.348
Density of PFS	1.00 (0.98–1.02)	0.985
Volume of Perfusion Deficit	1.03 (1.01–1.05)	0.002
Perfusion Deficit as a Percentage of Total Kidney Parenchyma	1.11 (1.05–1.17)	<0.001
CRP	1.11 (1.05–1.17)	<0.001
WBC	1.05 (0.99–1.11)	0.125
Procalcitonin	1.01 (0.98–1.05)	0.354
Age	1.03 (1.01–1.06)	0.011
Sex (male vs. female)	2.30 (0.90–5.86)	0.082
B. Multivariable logistic regression analysis		
Feature	OR (95% CI)	*p*-Value
Perfusion Deficit as a Percentage of Total Kidney Parenchyma	1.09 (1.00–1.18)	0.059
CRP	1.11 (1.03–1.20)	0.010
Age	1.06 (1.02–1.11)	0.002
Male sex	4.19 (1.04–16.90)	0.044

CI, confidence interval; CRP, C-reactive protein; LOS, length of hospital stay; OR, odds ratio; PFS, perirenal fat stranding; WBC, white blood cell. The odds ratio for CRP is expressed per 10 mg/L increase (equivalent to 1 mg/dL). Odds ratios for perfusion deficit percentage are expressed per 1 percentage-point increase.

## Data Availability

The data presented in this study are available on request from the corresponding author due to restrictions related to patient privacy and institutional policies.

## Abbreviations

The following abbreviations are used in this manuscript:

ACR	American College of Radiology
APN	Acute Pyelonephritis
CRP	C-reactive Protein
CT	Computed Tomography
LOS	Length of Hospital Stay
MRI	Magnetic Resonance Imaging
PFS	Perirenal Fat Stranding
ROI	Region of Interest
US	Ultrasound
UTI	Urinary Tract Infection
WBC	White Blood Cell
